# Healthcare‐Seeking Behaviors in a Depopulated Rural Area of Shiga Prefecture, Japan in 2025: A Descriptive Study Using the Ecology of Medical Care Model

**DOI:** 10.1002/jgf2.70169

**Published:** 2026-08-19

**Authors:** Yoshio Hisata, Sakiya Nishida, Risa H. Yoshinaga, Naoko E. Katsuki, Masaki Tago, Yuki Ueda, Takashi Sugioka, Masaki Amenomori, Katsumi Higashino, Yoshio Naya

**Affiliations:** ^1^ Department of Internal Medicine Nagahama City Kohoku Hospital Nagahama Japan; ^2^ Department of General Medicine Saga University Hospital Saga Japan; ^3^ Department of General Medicine Koka City Shigaraki Central Hospital Koka Japan; ^4^ Department of Internal Medicine Nishiazai Clinic Nagahama Japan; ^5^ Department of General Medicine Saga City Fuji‐Yamato Spa. Hospital Saga Japan; ^6^ Department of Family Medicine Shiga Center for Family Medicine Gamo‐gun Japan; ^7^ Department of Pediatrics Nagahama City Kohoku Hospital Nagahama Japan; ^8^ Department of Urology Nagahama City Kohoku Hospital Nagahama Japan

**Keywords:** ecology of medical care, health behavior, population decline, primary care, rural health

## Abstract

**Background:**

Japan will reach peak population aging around 2043. To better understand healthcare‐seeking behaviors in depopulated and aging communities, we examined healthcare utilization patterns in rural areas of Japan throughout 2025.

**Methods:**

Using the Ecology of Medical Care model, we conducted a descriptive study in a mountainous area of Japan. Health behaviors were summarized per 1000 population with 95% confidence intervals (CIs).

**Results:**

A total of 1285 residents reported 2021 health problems. The median age was 63 years (interquartile range, 39–74 years), and 47.2% of respondents were aged ≥ 65 years. Per 1000 population (95% CI), the estimated numbers of individuals were: no symptoms, 782 (759–805); observation, 20 (13–28); self‐care without medical consultation, 34 (24–43); clinic visits, 42 (31–53); primary hospital visits, 52 (40–64); secondary hospital visits, 31 (22–40); tertiary hospital visits, 8 (3–13); ambulance transport, 2 (0–5); hospitalization, 27 (18–36); and urgent home visits 2 (0–5). Self‐care without medical consultation among young adults as well as hospitalizations and ambulance transports among those aged ≥ 75 years were more frequent; children often used higher‐level hospital services.

**Conclusion:**

In rural mountainous communities, younger adults were more likely to rely on self‐care, whereas older adults had higher rates of hospitalization, and children used centralized hospitals more frequently. These findings provide insight into healthcare‐seeking patterns in communities experiencing population aging and decline.

## Graphical abstract

In a rural mountainous community with advanced aging (47.2% aged ≥ 65 years), diverging healthcare use patterns (164 visits per 1000 population) showed that children used centralized higher‐level hospitals, younger adults relied on self‐care, and older adults required more hospitalization.

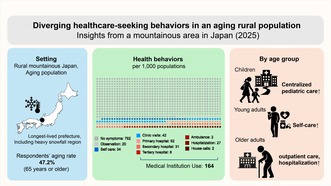

## 1 Introduction

The United Nations projects that by 2080, the global population aged ≥ 65 years will exceed that aged ≤ 20 years [1]. Japan is one of the longest‐living countries in the world; however, its total population has been declining, and the number of people aged ≥ 65 years is expected to peak in 2043 [2]. Depopulated areas in Japan are experiencing declining birth rates, population aging, and an overall population decline ahead of other regions of the country [3]. Therefore, analyzing health behaviors in depopulated areas is valuable for predicting near‐future medical care needs in Japan, and ultimately, in other aging societies worldwide.

The Ecology of Medical Care model was first proposed in 1961 as a conceptual framework for understanding the structure of healthcare utilization at the population level [4]; it was later revisited in 2001 [5]. The model describes residents' responses to health problems as a hierarchical structure comprising symptom experience, self‐care, outpatient visits, and hospitalization; it is useful for analyzing health‐seeking behaviors. Several studies applying the Ecology of Medical Care Model have been conducted in Japan, including nationwide studies [6] and subsequent re‐evaluations [7, 8]; studies focusing on children [9] and older adults [10], studies conducted on remote islands [11, 12]; and investigations before and after the COVID‐19 pandemic [13, 14]. Despite these investigations, evidence from mountainous rural areas is lacking. Rural mountainous areas account for 66.4% of Japan's land area [15]; this highlights the importance of understanding the role of healthcare as essential infrastructure in these regions.

This study aimed to describe the healthcare‐seeking behaviors in a mountainous rural area of Japan using the Ecology of Medical Care model and discuss the findings in the context of previous studies, thereby providing insights into healthcare utilization in depopulated and aging communities.

## 2 Methods

### 2.1 Research Design

This descriptive study was conducted based on the Ecology of Medical Care Model, using a self‐administered questionnaire. The study was conducted as a census survey of a geographically defined population using a mail‐based questionnaire.

### 2.2 Participants and Time Frame

The survey was conducted from July to September 2025 among 10,603 residents of 5164 households in Kinomoto town, Yogo town, and Nishiazai town in the northern area of Nagahama City, Shiga Prefecture.

### 2.3 Regional Characteristics

As of 2025, Shiga Prefecture ranks among the longest‐lived prefectures in Japan, with a life expectancy of 82.7 years for men (1st nationally) and 88.2 years for women (2nd nationally) [16]. According to the 2020 municipal life tables, life expectancy in Nagahama City was 82.2 years for men (301st of 1887 municipalities) and 88.2 years for women (247th of 1887 municipalities), placing it in the upper tier nationwide [17]. The overall disease burden in Shiga Prefecture is lower than the national average; for example, Shiga's age‐standardized death rates for cerebrovascular disease (37.9 per 100,000 people) are the lowest in Japan [18]. The three surveyed towns are located in an area with heavy snowfall. In 2025, when the survey was conducted, the baby boomer generation in Japan reached ≥ 75 years of age [19].

### 2.4 Japan's Healthcare System and the Medical Care Delivery System in the Study Area

Japan has adopted a system of universal health insurance and free access to medical institutions; this has yielded high levels of population health and equity at relatively low cost [20]. Unlike gatekeeping systems in many other countries, patients are not required to obtain referrals before accessing higher‐level care. In principle, patients may seek care 24 h a day at the primary (community‐based clinics and small hospitals), secondary (general hospitals), and tertiary (advanced acute care hospitals) levels; moreover, ambulance services are provided free of charge nationwide. At the time of the survey, patient copayments were 30% for non‐elderly adults and 10%–20% for older adults, depending on their income. Public subsidy programs further reduce the out‐of‐pocket costs for children, pregnant women, single‐parent households, individuals with severe disabilities, and some of the older adults (e.g., those living alone). While this program is generally means‐tested, a universal scheme without income restrictions has recently been applied for children. As a result, the financial barriers to care are relatively low. However, under this free‐access system, it is difficult to restrict patients from seeking higher‐level care beyond their medical needs. Therefore, measures such as ambulance user fees or additional charges for visits without referrals have been introduced to discourage the non‐urgent use of higher‐level facilities. In the study area, ambulance services have remained free, but secondary‐ and tertiary‐level hospitals charge additional fees from patients without a referral; this has discouraged non‐urgent visits.

### 2.5 Measurements

Data were collected using a self‐administered questionnaire. The participants were asked to report their age and sex as well as whether they had experienced any health problems within the past month; if so, the total number of such episodes. For each health problem, respondents were asked to indicate the type of healthcare‐seeking behavior they adopted by selecting all applicable options from the following ten categories:

1. No symptoms.
2. Self‐monitoring (waited and observed without seeking care).
3. Responses other than visiting a medical institution (e.g., consulting family members or acquaintances, searching the internet, using supplements or household medications, or using over‐the‐counter [OTC] drugs, corresponding to complementary and alternative medicine [CAM] care in previous studies).
4. Visit to a clinic.
5. Visit to a hospital within the primary healthcare service area.
6. Visit to a hospital within the secondary healthcare service area.
7. Visit to a hospital within the tertiary healthcare service area.
8. Transport via ambulance.
9. Hospitalization.
10. Receipt of a home visit by a physician.

In Japan, primary care services are provided not only by clinics, but also by small‐ and medium‐sized hospitals. Therefore, the primary care facilities included in this study included both clinics and hospital outpatient departments that provide first‐contact care.

Regarding healthcare utilization, the respondents were instructed to exclude regularly planned visits (Planned Clinic/Hospital Visits) and scheduled home medical care (Planned Home Visits) from their responses. For health problems occurring in minors, the head of the household reported the child's age and the number of episodes. Respondents were instructed not to include regular outpatient visits or scheduled home medical care. This exclusion was intended to avoid classifying residents with stable chronic conditions and no current symptoms as healthcare users, based solely on routine scheduled care.

### 2.6 Geographic Characteristics, Healthcare Structure, and Geospatial Mapping of the Study Area

**FIGURE 1:**
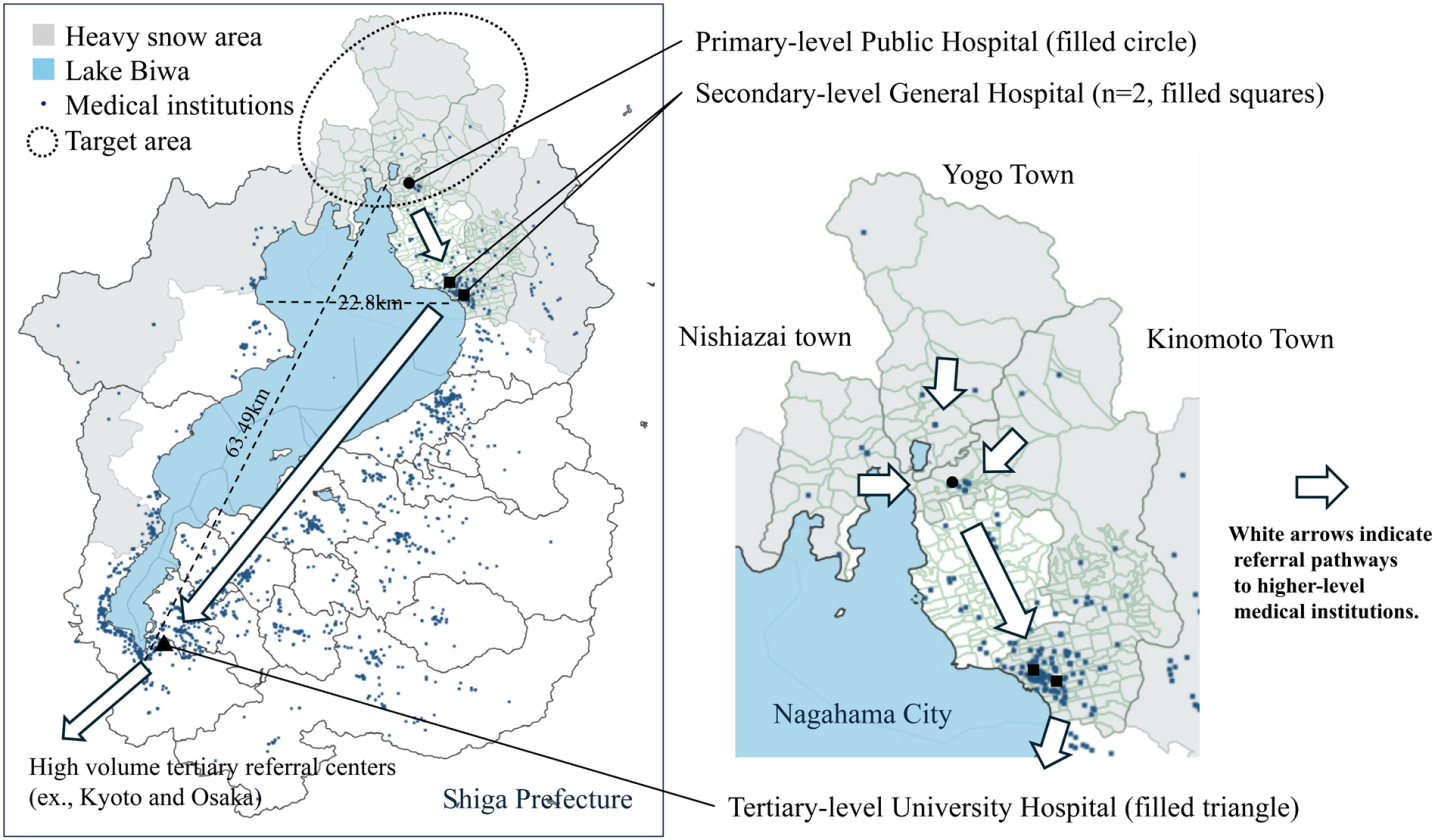
Geographical relationship between the study area and medical institutions. The study area comprised the towns of Kinomoto, Yogo, and Nishiazai in Nagahama City, located in northern Shiga Prefecture (dotted circles). The gray area in the north indicates a heavy snowfall region and the light blue area in the center represents Lake Biwa. Dark blue dots indicate medical institutions within the prefecture and all dots within the study area represent clinics. The filled circles indicate primary‐level public hospital, filled squares indicate secondary‐level general hospitals, and filled triangles indicate tertiary‐level university hospitals used by the residents of the study area. The white arrows represent the main referral routes from clinics to hospitals in the primary, secondary, and tertiary medical service areas. The dashed line indicates the distance across Lake Biwa.

Figure 1 illustrates the geographic layout of the study area. Data on designated heavy snowfall areas, lakes, marshes, and the locations of medical institutions [21] were processed using QGIS (QGIS Geographic Information System, QGIS Association, Zurich, Switzerland; version 3.30). One small public hospital (Primary‐level Public Hospital, filled circle) is located in the southern part of the study area and provides general internal medicine, emergency care, and inpatient care for minor specialties. Within a 15‐km radius, eight solo practice clinics (small blue dots) were distributed across the area, including five internal medicine clinics, one ophthalmology clinic, and two dental clinics. Most clinic physicians are elderly, and systems for nighttime home medical care, including home visits, are limited. No clinics specializing in pediatrics, orthopedics, dermatology, otolaryngology, psychiatry, or obstetrics and gynecology are available in the primary healthcare service area. In public hospitals, outpatient services are available during daytime, with some specialty clinics operating on a limited weekly basis. Internal medicine subspecialties and dialysis services are available, allowing for the management of certain advanced conditions. Part‐time physicians provide cardiology, pulmonology, and neurology services on a limited basis. For acute illnesses at night or on holidays, patients may visit public hospitals or clinics in the southern secondary healthcare service area; however, public transportation is unavailable after 8:00 p.m. owing to driver shortages. Patients transported via ambulance who cannot secure transportation to home may be admitted for short‐term observation. Two secondary‐level hospitals (filled squares) are located within 15 km south of the study area. Patients who require advanced treatment such as catheter‐based interventions, acute stroke management, and surgical procedures, are transferred accordingly. Access to tertiary‐level institutions (filled triangles) requires travel of approximately 100 km, and referrals to high‐volume centers may involve distances of 150–200 km to the south.

### 2.7 Statistical Analysis

Statistical analyses were conducted using the complete‐case approach. The unit of analysis was each episode of healthcare‐seeking behaviors in response to a health problem. Although individuals may report multiple episodes, intraindividual clustering was not considered because this study was descriptive. When multiple or overlapping episodes were reported within a category, each participant was counted once per health behavior. The highest category was used when the responses corresponded to more than one category (1–10), the highest category was used. To describe the population structure, the respondents were aggregated and a population pyramid was constructed. The frequency of health problems was tabulated according to healthcare‐seeking behaviors, and the proportions were calculated. The estimated number per 1000 population was obtained by multiplying the proportion by 1000. Ninety‐five percent confidence intervals (CIs) were calculated using the Wald method. The results are illustrated based on the ecology of the medical care model. Age‐stratified analyses were performed using the following categories: 0–19, 20–39, 40–64, 65–74, 75–84, and ≥ 85 years. All statistical analyses were performed using Stata/BE version 19.5 (StataCorp LLC, College Station, TX, USA).

### 2.8 Ethical Considerations

Submission of the questionnaire was considered as consent to participate. Participants were informed that their responses would be anonymized, that participation would be voluntary, and that they could withdraw at any time. When minors were included, consent was substituted with responses provided by the parent or guardian (head of household). This study was approved by the Ethics Committee of Nagahama City Kohoku Hospital (approval no. FY 2024‐02).

## 3 Results

Of the 10,603 eligible residents, 1285 were included in the analysis, a response rate of 12.1%. Among these respondents, 537 were men and 530 were women. The sex of 218 individuals aged < 20 years was unknown because the responses were provided by the household head. The respondents reported 2021 health problems (1.57 health problems per person per month). The median age was 63 years (interquartile range: 39–74 years) and the proportion of older adults (≥ 65 years) was 47.2%.

**FIGURE 2:**
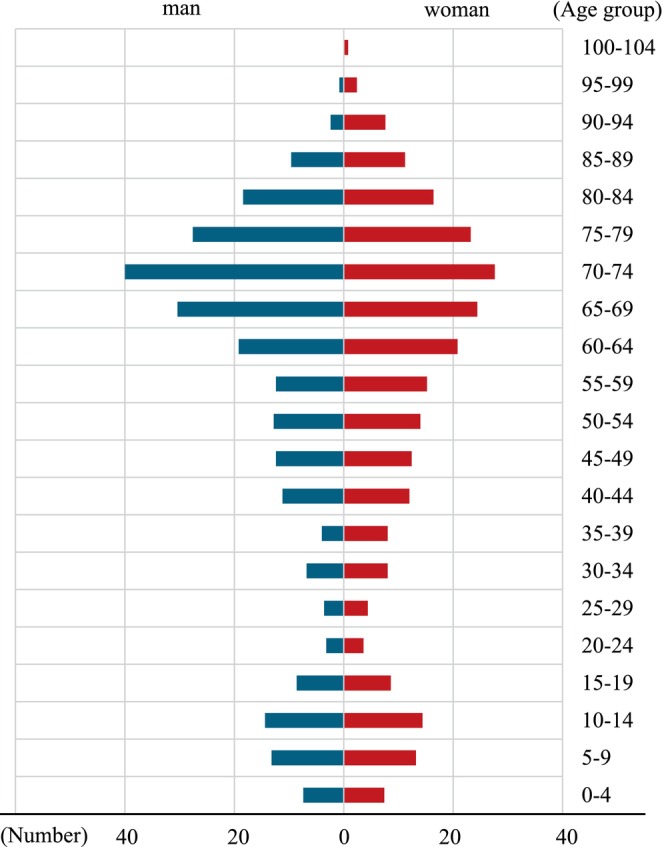
Population pyramid of the respondents. The distribution shows a bell‐shaped pattern, with the largest group in the early 70s. For individuals aged 0–19 years, sex was unknown; therefore, the number of respondents in each age group was equally divided between the sexes to construct the population pyramid.

The population pyramid is illustrated in Figure 2. It exhibited a bell‐shaped distribution, with the largest proportion in the 70‐ to 79‐year age group. The proportion of women was higher among those aged ≥ 85 years (Table S1).

Based on all reported episodes, the estimated number of individuals per 1000 population for each health‐seeking behavior (with 95% CIs) was as follows: no symptoms, 782 (734–831); watchful waiting, 283 (254–312); self‐care without visiting a medical institution, 123 (104–142); clinic visit, 91 (75–108); primary hospital visit, 153 (131–174); secondary hospital visit, 69 (55–84); tertiary hospital visit, 14 (8–21); ambulance transport, 18 (11–25); hospitalization, 37 (27–48); and home visit, 2 (1–7). The proportional distribution is illustrated in Figure S1. The distribution of the estimated number of episodes of individuals according to health behavior and their age‐specific breakdowns are shown in Table S2. The aggregate counts are presented in Table S3.

**FIGURE 3:**
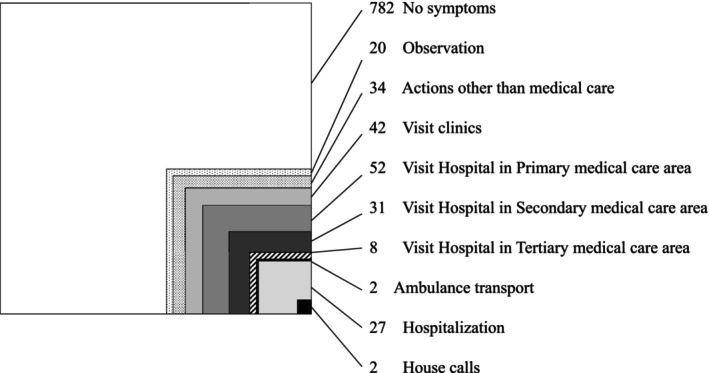
Distribution of health problems and healthcare‐seeking behaviors per 1000 population per month in three mountainous towns, including heavy snowfall areas, in Japan in 2025.

Table 2 shows the results after treating multiple responses from the same respondent as a single case and classifying episodes according to the highest level of care when multiple care levels were reported. The estimated number of response episodes per 1000 population was as follows: no symptoms, 782 (759–805); watchful waiting, 20 (13–28); self‐care without visiting a medical institution, 34 (24–43); clinic visit, 42 (31–53); primary hospital visit, 52 (40–64); secondary hospital visit, 31 (22–40); tertiary hospital visit, 8 (3–13); ambulance transport, 2 (0–5); hospitalization, 27 (18–36); and home visit, 2 (0–5). The proportional distribution is illustrated in Figure 3 and the aggregated counts are shown in Table S4.

Age‐specific distributions are also presented in Table 1 and Table S2 (the overview of these distributions was shown in Figure S2). Tertiary hospital visits were more frequent among individuals aged 0–19 and 20–39 years than in other age groups (4–18 individuals per group). Among those aged 40–64 years, self‐care without visiting a medical institution was relatively frequent. In individuals aged ≥ 65 years, the frequency of experiencing symptoms increased, and ambulance transport and hospitalization became more frequent in the older age groups. The use of home visits was observed only in the 40–64 and 65–74 years age groups.

**TABLE 1:** Distribution of each estimated population stratified by age, sex in Ecology of medical care model.

Category of health behavior	Age group						
	Total	0–19	20–39	40–64	65–74	75–84	Over 85
	Number/1000 (95% CI)						
No symptoms	782 (759–805)	830 (780–880)	838 (765–910)	817 (777–857)	752 (703–800)	701 (640–763)	760 (669–850)
Observation	20 (13–28)	19 (1–35)	10 (0–29)	22 (7–37)	23 (6–40)	23 (3–44)	11 (0–33)
Actions other than medical care	34 (24–43)	28 (6–49)	38 (1–76)	48 (25–70)	39 (18–61)	19 (1–37)	0 (0–0)
Visit clinics	42 (31–53)	64 (32–96)	38 (1–76)	37 (18–57)	36 (15–56)	37 (12–63)	46 (2–90)
Visit hospital in primary medical care area	52 (40–64)	18 (1–35)	19 (0–45)	31 (13–50)	69 (41–97)	107 (66–149)	69 (16–121)
Visit hospital in secondary medical care area	31 (22–40)	14 (0–30)	19 (0–45)	22 (7–37)	43 (20–64)	61 (29–94)	11 (0–34)
Visit hospital in tertiary medical care area	8 (3–13)	18 (1–35)	19 (0–45)	3 (0–8)	3 (0–9)	5 (0–14)	11 (0–33)
Ambulance transport	2 (0–5)	0 (0–0)	0 (0–0)	0 (0–0)	3 (0–9)	5 (0–14)	0 (0–0)
Hospitalization	27 (18–36)	9 (0–22)	19 (0–45)	14 (2–26)	29 (10–48)	42 (15–69)	92 (31–154)
House calls	2 (0–5)	0 (0–0)	0 (0–0)	6 (0–14)	3 (0–9)	0 (0–0)	0 (0–0)

The findings of these previous studies are presented in Table 2 (left). In a nationwide survey conducted in 2005, 862 individuals without symptoms were reported per 1000 population [6]. In the present study, conducted in a rural mountainous area in 2025, the corresponding figure was 782 per 1000 population. The 2005 survey reported that 49 individuals per 1000 population received care outside the healthcare system. The corresponding figure in the present study was 123 per 1000 population. The number of clinic visits was 307 per 1000 population in the 2005 survey and 91 per 1000 population in the present study. For outpatient hospital visits, the 2005 survey reported 88 visits per 1000 population at the primary‐/secondary‐level hospitals and 6 per 1000 population at the tertiary‐level hospitals. In the present study, the corresponding figures were 153, 69, and 14 per 1000 population in the primary‐, secondary‐, and tertiary‐level hospitals, respectively.

**TABLE 2:** Comparison of this study with previous studies.

Category about health behavior	Characteristics of the study			
	The present study (*n* = 1285)	20 years ago^a^ (*n* = 3477)	The present study (under 20 years old, *n* = 218)	Study in child^b^ (*n* = 1024)
	Episodes number/1000 people			
No symptoms	782	862	830	872
Observation	283	NA	211	NA
Actions other than medical care	123	49	55	NA
Visit clinics	91	307	83	335
Visit hospital in primary medical care area	153	88	41	82
Visit hospital in secondary medical care area	69		50	
Visit hospital in tertiary medical care area	14	6	32	2
Ambulance transports	18	10	5	21
Hospitalizations	37	7	9	2
Urgent home visits	2	3	0	4

^a^ Fukui et al. [6].

^b^ Ishida et al. [9].

The findings from the 2012 survey on children's healthcare‐seeking behaviors and those observed among children in the present study are shown in Table 2 (right). The number of clinic visits was 335 per 1000 population in the 2012 survey and 83 per 1000 population in the present study. Hospital visits in the 2012 survey were reported to be 82 per 1000 population for primary‐/secondary‐level hospitals and 2 per 1000 population for tertiary‐level hospitals. In the present study, hospital visits were reported at 41, 50, and 32 visits per 1000 population in primary‐, secondary‐, and tertiary‐level hospitals, respectively. Ambulance use was reported at 21 per 1000 population in the 2012 survey and 5 per 1000 population in the present study.

## 4 Discussion

This study describes the ecology of medical care in a rural mountainous area of Japan in 2025, when the baby boomer generation reached the age of ≥ 75 years [19]. The study area had a high aging rate (47.2%). Our findings provide insight into healthcare utilization patterns in communities experiencing advanced population aging. Although the overall hierarchical structure was similar to those reported in previous studies, several changes were observed at each level. These changes may be discussed from four perspectives: (1) characteristics of self‐care behaviors, (2) healthcare utilization in the context of population aging, (3) patterns of pediatric healthcare utilization associated with declining birth rates, and (4) interpretation of the findings in relation to previous domestic and international studies.

### 4.1 Characteristics of Self‐Care Behavior

According to previous domestic Ecology of Medical Care studies [6, 7, 8], the reported use of CAM is 49 per 1000 population. In the present study, the corresponding number was 123 per 1000 population (Table 2). This observation may be viewed in the context of broader changes in the access to health information. Over the past two decades, access to health information through the internet and social media has expanded substantially in Japan. An internet‐based survey reported that 30%–50% of respondents use online health information for decision‐making [22], and social media use has been associated with improved self‐care efficacy [23]. According to age group, care other than visiting medical institutions was more frequent than the overall average among those aged 20–39, 40–64, and 65–74 years, suggesting that self‐management of minor health problems is relatively common. However, because internet use and health literacy were not directly measured in this study, the factors underlying this pattern remain unclear.

### 4.2 Healthcare Utilization in the Context of Population Aging

In this study, healthcare utilization was characterized by relatively frequent hospital visits and hospitalizations. Previous domestic studies have reported 7 hospitalizations per 1000 population [6]. In the present study, 37 hospitalizations per 1000 population were observed. The hospitalization rate in the present study appeared numerically higher than that reported in previous domestic studies. However, these findings should be interpreted cautiously because of differences in study populations and settings. A study from the United Kingdom reported increases in multimorbidity and frailty syndrome among hospitalized patients [24], suggesting that population aging may be associated with changing care needs. Previous domestic studies have reported 88 hospital visits within a primary care area per 1000 population. In the present study, 153 hospital visits per 1000 population were observed. These findings can be interpreted in the context of advanced aging of a population. Emergency transport and hospitalization were more frequent among those aged ≥ 75 years. Although this pattern may be consistent with previous findings regarding on ambulatory care sensitive conditions (ACSCs), the present study did not collect diagnoses or causes of hospitalization. Therefore, the relationship between the observed hospitalizations and ACSCs should be interpreted with caution. Previous studies have shown that social support such as having a spouse or nearby children is associated with lower rates of emergency hospitalization [25]. However, in rural mountainous areas with declining populations, older adults often live alone and far from their families, highlighting the importance of social support systems in preventing ACSC‐related hospitalizations. Municipalities with more home care resources had lower ACSC hospitalization rates [26]. Previous domestic studies have reported 2 home visits per 1000 population, whereas the corresponding figure in the present study was 3 per 1000 population. Disparities in the availability of home medical care systems, including home‐based emergency care, have been reported in rural areas [27], indicating an important challenge for future healthcare systems.

### 4.3 Patterns of Pediatric Healthcare Utilization

Previous studies have reported that children have higher healthcare utilization rates than adults [9]. However, in the present study, clinic visits among children were relatively low, whereas hospital visits were more common. This pattern may reflect the absence of pediatric clinics in the study area (see Section 2) and national and prefectural policies to consolidate pediatric emergency care resources in response to declining birth rates [28, 29]. These findings reflect the limited availability of pediatric care at the clinic level within the primary care area. Although board‐certified family physicians receive training in pediatric care, the practical implementation remains limited [30]. Strengthening pediatric care in primary care settings could help improve geographic access to care and reduce the burden on higher‐level medical institutions.

### 4.4 Interpretation of the Findings in Relation to Previous Studies in Japan

Studies conducted during the COVID‐19 pandemic in 2022 [13] and a post‐pandemic reassessment in 2025 [14] reported that healthcare visits declined during the pandemic and did not fully return to pre‐pandemic levels. In the present study, 283 per 1000 population reported watchful waiting despite experiencing symptoms. Limited access to medical services and reduced avoidance of healthcare visits may be involved; however, further investigation is required. Studies conducted in other remote areas reported fewer hospital visits on small remote islands [11, 12]. These differences may reflect variations in the availability of healthcare resources compared to the availability in rural mountainous areas.

### 4.5 Interpretation of the Findings in Relation to International Studies

In the following discussion, direct comparisons between our findings and those of other countries should be interpreted with caution, as healthcare systems and regional characteristics vary substantially across countries.

Several characteristics of Japan's healthcare system and health‐seeking behaviors can be identified by referring to the ecology of medical care studies conducted in other countries. Reports from the United States in 1961 [4] and 2001 [5] documented fewer healthcare visits than those observed in this study.

Studies conducted in Beijing [31] and Shanghai [32] reported a notable proportion of visits to traditional Chinese medical practitioners, a feature that is not observed in Japan. In South Korea, the use of tertiary care institutions is reported to be relatively high (35 per 1000 population) [33]; a similar pattern was observed in the present study.

In contrast, Switzerland has a registered family physician system, and the use of CAM is relatively low (6 per 1000 population) [34]. Although direct comparisons should be interpreted with caution, these findings provide insights into the potential role of primary care physicians in guiding healthcare‐seeking behaviors.

A study conducted in France reported that consultations with general practitioners increased with age [35]. Similarly, in this study, the proportion of episodes with reported symptoms was higher among older adults, suggesting similar care needs. These findings are consistent with those of our previous study conducted in the same region, which showed that a richer primary care experience among residents with chronic conditions was associated with better self‐rated health [36]. The expanding role of board‐certified family physicians, whose training has recently been promoted in Japan [37], may therefore be important in addressing these healthcare needs.

Studies conducted in the rural areas of other countries have also reported characteristics suggestive of limited access to healthcare services. A study from Switzerland reported that rural residents were less likely than urban residents to report health problems, seek medical advice, or visit general practitioners [34]. Although direct international comparisons should be interpreted with caution, these findings are consistent with the possibility that residents of rural mountainous areas face greater barriers to accessing healthcare than those living in urban settings.

### 4.6 Limitations

This study has several limitations. First, this study was conducted in a single geographically limited area with unique characteristics, including heavy snowfall, limited public transportation, a shortage of specialists, and advanced population aging, which may limit the generalizability of the findings to other rural areas in Japan. The distribution of medical specialties within primary care areas varies according to region and may influence healthcare utilization patterns. Additionally, the response rate was relatively low (12.1%), raising the possibility of selection bias. Respondents may have been older, more health‐conscious, and more likely to utilize healthcare services than non‐respondents, which may have resulted in an overestimation of healthcare utilization patterns. Second, home medical care utilization may have been underestimated, as home visit users often have difficulty responding to questionnaires and proxy responses from family members may be limited. Third, regular outpatient visits and scheduled home medical care were excluded from the assessment of healthcare utilization. Therefore, while the study provides insight into health‐seeking behavior in response to symptoms, it may not fully reflect healthcare needs among individuals with stable chronic conditions. Fourth, sex‐related information for minors was unavailable despite known age‐related differences in healthcare utilization according to sex [38]. Finally, the survey was conducted between July and September and did not capture seasonal variations, including snow‐related barriers to healthcare access in regions with heavy snowfall.

## 5 Conclusion

This study visualized healthcare‐seeking behaviors among the residents of a rural mountainous region using the Ecology of Medical Care model. In the context of previous domestic studies, the findings suggest differences in the structure of healthcare utilization, including greater use of self‐care and CAM as well as higher rates of hospital visits and hospitalizations. These patterns may be associated with population aging and structural changes in the regional healthcare resources. In addition, pediatric healthcare‐seeking behavior showed fewer clinic visits and more hospital visits, which may be related to the characteristics of the local healthcare delivery system and demographic changes, including declining birth rates. Among older adults, the relatively high frequency of symptom experiences and hospitalizations suggests an evolution of community care needs. Overall, the findings provide an overview of healthcare utilization in rural mountainous areas and highlight the importance of strengthening primary care and home healthcare systems as well as developing healthcare delivery systems that reflect regional characteristics.

## Author Contributions

**Yoshio Hisata:** conceptualization, investigation, methodology, software, formal analysis, data curation, writing – review and editing, writing – original draft, funding acquisition, visualization, project administration, resources. **Yoshio Naya:** investigation, supervision, project administration. **Takashi Sugioka:** supervision. **Yuki Ueda:** investigation, conceptualization. **Naoko E. Katsuki:** supervision, formal analysis, writing – review and editing. **Masaki Amenomori:** investigation, supervision. **Katsumi Higashino:** investigation, project administration. **Masaki Tago:** supervision, project administration, formal analysis. **Risa H. Yoshinaga:** supervision, formal analysis. **Sakiya Nishida:** methodology, supervision, conceptualization, project administration, writing – review and editing.

## Funding

Yoshio Hisata received a NISSAY Foundation Grant for Practical Research from the Aged Society in 2024.

## Ethics Statement

This study was approved by the Ethics Committee of Nagahama City Kohoku Hospital (approval no. FY 2024‐02).

## Consent

Consent was obtained after clearly stating that the participants were free to respond or withdraw their responses.

## Conflicts of Interest

The authors declare no conflicts of interest.

## Supporting information


**Figure S1:** Distribution of episodes health problems and healthcare‐seeking behaviors per 1000 population per month. In contrast to Figure 3, all episodes reported by individual respondents were included in the analysis, including multiple episodes reported by the same respondent.
**Figure S2:** Overview of the age‐specific distribution of numbers and episodes of health problems and healthcare‐seeking behaviors per 1000 population. The upper panel shows the distribution of number of individuals, and the lower panel shows the distribution of episodes. Comparing the two panels illustrates the increasing frequency of healthcare utilization with age and the occurrence of multiple healthcare‐seeking behaviors among individuals. In conjunction with Figure 3 and Figure S1, this figure provides an overview of age‐related patterns in healthcare‐seeking behaviors among children, young adults, and older adults.

**Table S1:** Characteristics of the respondents.
**Table S2:** Distribution of each episodes stratified by age, sex.
**Table S3:** Responded episodes' number of the Table S1.
**Table S4:** Respondents' number of the Table 2.

## Data Availability

The data supporting the findings of this study are available from the corresponding author, Yoshio Hisata, upon reasonable request.
